# Immunoprofiling of human uterine mast cells identifies three phenotypes and expression of ERβ and glucocorticoid receptor

**DOI:** 10.12688/f1000research.11432.2

**Published:** 2017-06-22

**Authors:** Bianca De Leo, Arantza Esnal-Zufiaurre, Frances Collins, Hilary O.D. Critchley, Philippa T.K. Saunders

**Affiliations:** 1 MRC Centres for Inflammation Research and Reproductive Health, The University of Edinburgh, Edinburgh, EH16 4TJ, UK; 2 MRC Centre for Inflammation Research, The University of Edinburgh, Edinburgh, EH16 4TJ, UK; 3 MRC Centre for Reproductive Health, The University of Edinburgh, Edinburgh, EH16 4TJ, UK

**Keywords:** chymase, tryptase, oestrogen, steroid receptor, ERb, GR

## Abstract

**Background:** Human mast cells (MCs) are long-lived tissue-resident immune cells characterised by granules containing the proteases chymase and/or tryptase. Their phenotype is modulated by their tissue microenvironment. The human uterus has an outer muscular layer (the myometrium) surrounding the endometrium, both of which play an important role in supporting a pregnancy. The endometrium is a sex steroid target tissue consisting of epithelial cells (luminal, glandular) surrounded by a multicellular stroma, with the latter containing an extensive vascular compartment as well as fluctuating populations of immune cells that play an important role in regulating tissue function. The role of MCs in the human uterus is poorly understood with little known about their regulation or the impact of steroids on their differentiation status.

The current study had two aims: 1) To investigate the spatial and temporal location of uterine MCs and determine their phenotype; 2) To determine whether MCs express receptors for steroids implicated in uterine function, including oestrogen (ERα, ERβ), progesterone (PR) and glucocorticoids (GR).

**Methods:** Tissue samples from women (n=46) were used for RNA extraction (n=26) or fixed (n=20) for immunohistochemistry.

**Results:** Messenger RNAs encoded by *TPSAB1* (tryptase) and *CMA1* (chymase) were detected in endometrial tissue homogenates. Immunohistochemistry revealed the relative abundance of tryptase MCs was myometrium>basal endometrium>functional endometrium. We show for the first time that uterine MCs are predominantly of the classical MC subtypes: (positive, +; negative, -) tryptase+/chymase- and tryptase+/chymase+, but a third subtype was also identified (tryptase-/chymase+). Tryptase+ MCs were of an ERβ+/ERα-/PR-/GR+ phenotype mirroring other uterine immune cell populations, including natural killer cells.

**Conclusions:** Endometrial tissue resident immune MCs have three protease-specific phenotypes. Expression of both ERβ and GR in MCs mirrors that of other immune cells in the endometrium and suggests that MC function may be altered by the local steroid microenvironment.

## Introduction

Mast cells (MCs) are long-lived tissue resident immune cells, derived from CD34 ^+^/c-kit ^+^ pluripotent progenitors, that reside in the bone marrow ( Kirshenbaum *et al*., 1999). MC progenitors are recruited into peripheral tissues by chemokines secreted by stromal cells, which together with stem cell factor, a complex array of cytokines, and a range of micro-environmental factors, are reported to stimulate the development of tissue resident mature MCs ( Valent *et al*., 1992). Mature MCs are usually classified according to the presence of one or more serine proteases (tryptase and/or chymase) in prominent cytoplasmic granules.

MCs are typically phenotyped as MC _TC_, with granules containing both tryptase (TPSAB1) and chymase (CMA1), or MC _T_, with granules only containing tryptase alone ( Collington *et al*., 2011; Wernersson & Pejler, 2014). It has been reported that MCs maturing in different tissue microenvironments can vary widely in the amount of tryptase and chymase they contain ( Caughey, 2007). When MCs are activated they de-granulate by exocytosis, releasing these serine proteases together with other inflammatory meditators ( Lorentz *et al*., 2012; Tiwari *et al*., 2008). The female sex hormones, oestradiol and progesterone, are thought to have an impact on MCs because many pathophysiological conditions attributed to MC activity have a higher prevalence in females than males ( Narita *et al*., 2007). Studies in non-reproductive tissue systems and those using the HMC-1 cell line (human MC line, ( Butterfield *et al*., 1988)) have reported that MCs express the oestrogen receptor α isoform (ERα) and progesterone receptor (PR) ( Jensen *et al*., 2010; Nicovani & Rudolph, 2002; Zaitsu *et al*., 2007). Some authors found evidence that MCs can be rapidly stimulated to degranulate by oestradiol via ERα ( Zaitsu *et al*., 2007). Glucocorticoid treatments are reported to reduce the number of tissue resident MCs by reducing concentrations of stem cell factor that are required for MC survival ( Finotto *et al*., 1997). Glucocorticoids are also reported to prevent MC activation via their high-affinity IgE receptor ( Smith *et al*., 2002).

The human endometrium undergoes physiological cycles of cellular proliferation, differentiation and secretory activity during each menstrual cycle ( Johannisson *et al*., 1987). In the absence of embryo implantation, the upper functional layer of the endometrium breaks down and is shed at menstruation, which is considered to bear the hallmarks of an inflammatory process ( Jabbour *et al*., 2006). Endometrial tissue adjacent to the myometrium (basal compartment) is not shed during menstruation and is implicated in the rapid re-epithelialisation, cessation of bleeding and restoration of tissue homeostasis facilitating regeneration of the functional layer. This monthly tissue remodelling is regulated by changes in cyclical ovarian steroid hormones with oestrogen increasing cell proliferation, progesterone inducing functional maturation of stromal cells in preparation for implantation, and progesterone withdrawal precipitating a cascade of changes culminating in tissue breakdown and menstruation ( Kelly *et al*., 2001; Maybin & Critchley, 2015; Salamonsen & Lathbury, 2000). Endometrial tissue contains stromal, epithelial, and endothelial cells, as well as a diverse population of immune cells, the most abundant of which are uterine natural killer cells (uNK) and macrophages ( Evans & Salamonsen, 2012; Thiruchelvam *et al*., 2013). We, and others, have investigated the impact of steroids on uNK cells and macrophages and shown that they contain both receptors that can bind oestrogens (ERβ isoform) and glucocorticoids ( Bombail *et al*., 2008; Henderson *et al*., 2003), but are immmuno-negative for ERα and PR. The concentrations of steroids in endometrial tissue are subject to local modulation by enzymes that metabolise sex steroids (androgens, oestrogens), as well as glucocorticoids ( Bamberger *et al*., 2001; Gibson *et al*., 2013; Gibson *et al*., 2016; McDonald *et al.*, 2006). The creation of a steroid rich microenvironment has an impact on the function of immune cells and the vasculature. For example, exposure of uNK to oestrogens increases their secretion of CCL2, which has an impact on vascular endothelial cells ( Gibson *et al*., 2015), and likewise exposure of macrophages to cortisol results in the release of factors that induce altered endometrial endothelial cell expression of angiogenic genes ( Thiruchelvam *et al*., 2016).

The basal portion of the endometrium sits directly on the myometrium, which is made up of three layers consisting of smooth muscle fibres and associated vasculature and stroma. The inner layer, adjacent to the endometrium, is also known as the junctional zone. This zone has circular muscle fibres and like the endometrium it is derived from the Mullerian duct ( Uduwela *et al*., 2000), whilst the other layers develop from non-Mullerian tissue. The smooth muscle cells of the myometrium (myocytes) are active during the non-pregnant menstrual cycle, with uterine peristalsis constituting one of their fundamental functions ( Kunz & Leyendecker, 2002). Like the endometrium, the myometrium is a steroid target organ with myocytes expressing receptors for oestrogens, progestagens and androgens, all of which can induce changes in gene expression ( Chandran *et al*., 2016; Makieva *et al*., 2016).

MCs have been identified in the human uterus, with reports that their phenotype is similar to lung MCs in terms of a response to secretagogues and release of prostaglandins, but a granule phenotype distinct to that of gut MCs ( Massey *et al*., 1991). There has also been interest in the role played by MCs in myometrial contractions and in fibroids, although whether they play an important role in either has been questioned ( Garfield *et al*., 2006; Menzies *et al*., 2011; Protic *et al*., 2016). A detailed study on uterine MCs was published by Jeziorska *et al*. (1995), who used immunohistochemistry to identify tryptase and chymase positive cells in 107 uterine samples taken across the menstrual cycle. They reported that there were similar MC numbers throughout the functional, basal layers of the endometrium, and myometrium during the menstrual cycle. In a smaller study Mori *et al.* (1997) immunostained uterine samples from 24 women with a variety of gynaecological disorders using antibodies against tryptase and chymase. They reported that the MCs were most abundant in the inner half of the myometrium and most endometrial MCs were of the MC _T_ phenotype.

In summary, the role of MCs in the human uterus is poorly understood and little is known about their regulation, or the impact of steroids within the uterine microenvironment on their differentiation status. The current study used tissue sections of human uterus to define the spatial and temporal location of MCs in the myometrium and endometrium, and explored their phenotype by examining the pattern of expression of the proteases tryptase and chymase using fluorescent co-staining. We also examined expression receptors for oestrogen (ERα, ERβ), progesterone (PR) or glucocorticoids (GR) to determine their ability to respond directly to steroids.

## Methods

### Patients and tissue recovery

Ethical committee approval was obtained from the Lothian Research Ethics Committee (LREC; approval numbers, 10/S1402/59 and 16/ES/0007). Patients were recruited by dedicated research nurses from clinics treating women for benign gynaecological conditions, including heavy menstrual bleeding and fibroids. In all cases written patient consent was obtained prior to tissue collection. Full details of patients are provided in Supplementary Table 1. The total number of women from which samples were obtained was 46. However, due to limited amount of tissue available, some samples were either fixed (n=20) or used for RNA extraction (n=26), but not for both. Patients were aged between 25–50 years (average of 39.8 years), reported regular menstrual cycles and had not taken any exogenous hormones in the three months prior to surgery. Stage of the menstrual cycle was evaluated by analysis of circulating steroid concentrations (P _4_, E _2_) using blood samples obtained at the time of surgery. Assays were performed by the Specialist Assay Service (Surf Facility, University of Edinburgh) and cycle stage was further confirmed by examination of tissue sections by an expert pathologist, Professor A.R.W. Williams (NHS, Royal Infirmary, Edinburgh). Critical inclusion criteria were the absence of pelvic pain, such as dysmenorrhea, absence of fibroids or presence of small fibroids only (<3 cm): none of these women had a diagnosis of endometriosis.

### RNA extraction, cDNA synthesis and qRT-PCR

Total RNA was extracted using the RNeasy Mini Kit (Qiagen, UK), according to manufacturer’s instructions. RNA concentration and purity was measured using the Nanodrop (LabTech International, UK) and standardised to 100ng/µl for all samples. Reverse transcription was performed using 100ng of RNA with 0.125× Superscript Enzyme in 1× VILO reaction mix (Thermo Fisher Scientific, UK) at 25°C for 10 minutes, followed by 42°C for 60 minutes and finally 85°C for 5 minutes. Quantitative PCR was performed using FAM labelled probes for *TPSAB1* (number 20) and *CMA1* (number 81) from the Universal Probe Library (Roche Diagnostics, UK) and VIC labelled human PPIA (Cyclophilin A) endogenous control (Thermo Fisher Scientific), with specific primers for *TPSAB1* (forward 5’-cctgcctcagagaccttcc-3’; reverse 5’-acctgcttcagaggaaatgg-3’) and *CMA1* (forward 5’-ttcacccgaatctcccatta-3’; reverse 5’-tcaggatccaggattaatttgc-3’) (Eurofins Genomics, UK). Primers directed against human cyclophillin A (CYC, PPIA) served as an endogenous control were supplied in a premade kit purchased from Thermo Fisher Scientific (catalogue number 4310883E). Each 15μl reaction consisted of 1μl of cDNA in 1× Express qPCR Supermix (Thermo Fisher Scientific) with 200nM of forward and reverse primer, 100nM probe, amplified for 40 cycles at 95°C for 15s followed by 60°C for 1 minute using the ABI Prism 7900HT Fast Real-Time PCR System (Applied Biosystems, UK). Analysis was by relative standard curve according to the recommendations of Bustin *et al.* (2009) using tonsil mRNA (ASD-0088; Applied StemCell, USA), chosen because it is a well-established positive control for mRNA expression of MC proteases ( Irani *et al.*, 1986).

Statistical analysis was carried out using GraphPad Prism 6.0 (GraphPad Software, USA). Data are presented as the median. One-way ANOVA was used, and Kruskal-Wallis was performed as a secondary test with Dunn’s multiple comparisons test. Criterion for significance was p<0.05.

### Phenotyping mast cells by immunohistochemistry and dual colour immunofluorescence

Immunohistochemistry was carried out on “full thickness” (uterine lumen to endometrial-myometrial junction) human uterine sections to localize MCs to the different tissue layers: myometrium, basal endometrium and functional (luminal) endometrium. Uterine biopsies were fixed in 4% neutral buffered formalin, embedded in paraffin wax and cut to 5μm sections. Following dewaxing and rehydration, sections were blocked in methanol peroxide for 30 minutes on a rocker at room temperature (RT), followed by 30 minutes blocking in normal goat blocking serum (Sigma Aldrich, Dorset, UK), before primary antibody incubation at 4°C overnight (16h): Tryptase, rabbit monoclonal, Abcam, UK; Chymase, mouse monoclonal, AbSerotec, UK; ERα, mouse monoclonal, Vector Laboratories, UK; ERβ mouse monoclonal, AbSerotec, UK. After washing in 1× Tris Buffered Saline + 0.05% Tween, slides were incubated with secondary antibody for 1 hour at RT, followed by 1:50 tyramide signal amplification (TSA Fluorescein Tyramide Reagent Pack, PerkinElmer, USA) for 10 minutes. Antigen retrieval was performed at pH6 in citrate buffer, and then further blocked with serum to avoid cross-reactivity. The second primary antibody was then added and incubated overnight at 4°C. Incubation with an appropriate secondary antibody at 4°C for 16 hours and a further TSA amplification step were carried out before counterstaining the sections with DAPI (1:500 dilution in TBS) and mounting with permafluor (ThermoFisher Scientific). Fluorescent images were acquired with a Zeiss Axioscan Z1 or a Zeiss 710 confocal microscope, and analysed with Zen Blue or Black software (version 2; Zeiss, Jena, Germany). Full antibody details can be found in Supplementary Table 2.

## Results

### Messenger RNAs encoding mast cell proteases were detected in human endometrium

**Figure 1.**
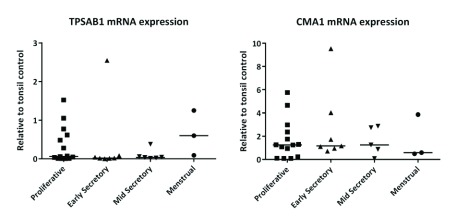
Messenger RNAs encoded by genes encoding mast cell proteases were not cycle stage dependent. ( **A**) *TPSAB1* mRNA and ( **B**) *CMA1* mRNA. Single dots represent different patient samples, and data are expressed as the median. Proliferative phase n=14, early secretory phase n=7, mid secretory phase n=6, and menstrual phase n=3.

In tissue homogenates of endometrium, total concentrations of messenger RNAs encoded by *TPSAB1* (gene for tryptase α and βisoforms; Figure 1A) and *CMA1* (chymase; Figure 1B) did not change significantly ( *TPSAB1* 0.254; *CMA1* 0.867), according to stage of the menstrual cycle. 

### Immunoflourescence identified three distinct mast cell subtypes in uterine biopsies collected during different stages of the menstrual cycle

**Figure 2.**
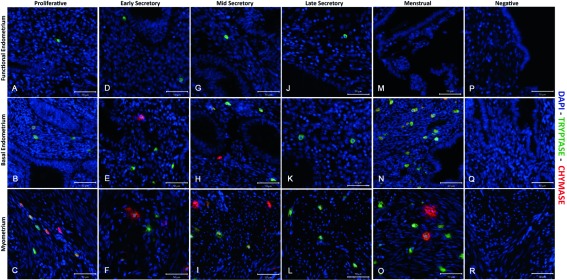
Comparison of immunoexpression of chymase and tryptase in “full thickness” (uterine lumen to endometrial/myometrial junction) tissue samples obtained from across the menstrual cycle. Note that mast cells were less abundant in the functional layer and appeared to be exclusively tryptase+/chymase-. ( **A**– **C**) Functional, basal endometrium and myometrium during proliferative phase (P); ( **D**– **F**) Early secretory phase (ES); ( **G**– **I**) Mid secretory phase (MS); ( **J**– **L**) Late Secretory phase (LS); ( **M**– **O**) Menstrual phase (M); ( **P**– **R**) Negative control (omission of primary antibody). Double immunofluorescence has revealed the presence of three uterine mast cell subtypes, single tryptase, single chymase and double tryptase-chymase positive cells. (P n=4, ES n=4, MS n=2, LS n=3, M n=2): negative controls were included on all sections.

**Figure 3.**
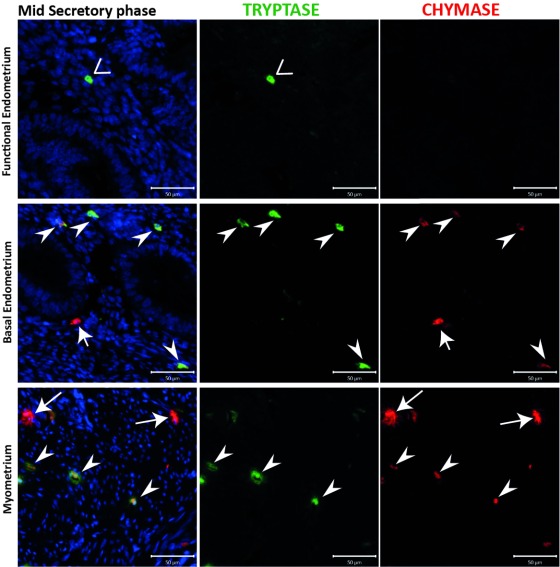
A population of chymase positive mast cells that did not express tryptase was identified in uterine tissue samples from the mid secretory phase. The endometrial compartment shows three different mast cell (MC) subtypes: tryptase single positive, chymase single positive and tryptase and chymase double positive. Basal endometrial MCs are chymase+/tryptase- single positive and double positive, instead functional endometrium MCs are fewer in number and show a chymase-/tryptase+ phenotype. MCs during the secretory appeared to be activated in the myometrium, releasing both proteases from the cytoplasm. (n=3) (Arrowheads: MC _TC_ cells; Vs: MC _T_ cells; arrows: MC _C_ cells).

**Figure 4.**
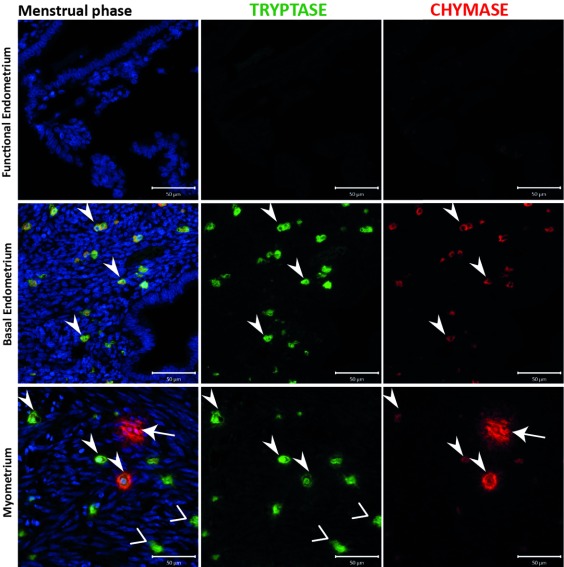
Samples from the menstrual phase contained chymase positive cells that appeared to be ‘activated’, as well as abundant double positive cells in the basal endometrium. Uterine mast cells (MCs) appear to be tryptase and chymase double positive, in both myometrial and basal endometrial layers, with a small portion of chymase+ MCs in the myometrium. MCs were not detectable in the functional layer. MCs during the menstrual phase appeared to be activated only in myometrial compartment, releasing both tryptase and chymase from the cytoplasm. In the basal endometrium, MCs showed a steady state instead. (n=2) (Arrowheads: MC _TC_ cells; Vs: MC _T_ cells; arrows: MC _C_ cells).

Immunoexpression of both tryptase and chymase positive cells were identified in all three layers of the human uterus examined in this study. In line with a previous report ( Jeziorska *et al*., 1995), the numbers of tryptase immunopositive cells appeared higher in the myometrium and adjacent basal layer of the endometrium than in the functional layer (green cells) in all phases of the cycle ( Figure 2 and Figure 3, Supplementary Figure 1– Supplementary Figure 3). Notably some of the chymase cells (red staining) in the myometrium appeared to be ‘activated’, with immunopositive staining being intense and diffuse within the tissue during both the early ( Supplementary Figure 2) mid ( Figure 3, arrow) and late ( Supplementary Figure 3) secretory and menstrual ( Figure 4, arrows) phases.

Examination of tissues from 20 of the patients obtained at different stages of the cycle, including the proliferative ( Supplementary Figure 1), early ( Supplementary Figure 2) mid ( Figure 3) and late ( Supplementary Figure 3) secretory and menstrual ( Figure 4) phases, also identified a population of MCs that were chymase positive, but without co-incident expression of tryptase (arrows). These cells appeared less abundant than those that were immunopositive for both tryptase and chymase (arrowheads), and were confined to the basal compartment of the endometrium and the myometrium.

The data obtained from immunohistochemical analysis of the 20 patients are summarized in Table 1.

**Table 1.** Summary of uterine mast cell phenotypes documented in tissue samples collected across the menstrual cycle.

		Proliferative	Early secretory	Mid secretory	Late secretory	Menstrual
**Mast cell** **sub-types**	**Endo**	MC _T_, MC _TC_	MC _T_, MC _TC_	MC _T_, MC _TC_, MC _C_	MC _T_	MC _TC_
	**Myo**	MC _TC_, MC _C_	MC _T_, MC _TC_, MC _C_	MC _T_, MC _TC_	MC _TC_	MC _TC_, MC _C_

### Tryptase-positive uterine mast cells were immunopositive for oestrogen receptor beta but did not express either oestrogen receptor alpha or progesterone receptor

**Figure 5.**
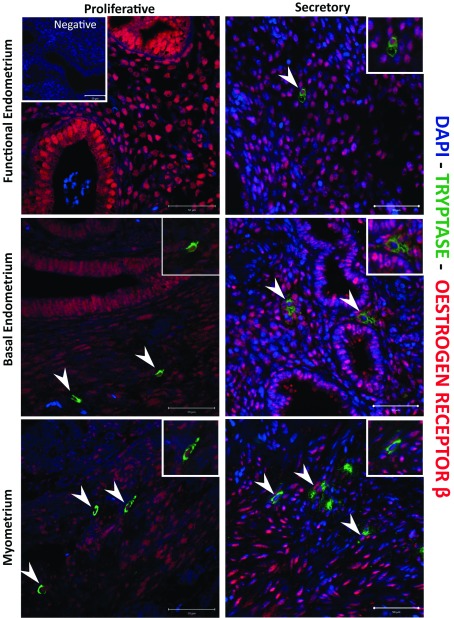
Mast cells are immunopositive for ERβ. Immunohistochemistry showed co-localization of ERβ in uterine mast cells (MCs). Nuclear expression of ERβ receptor (red staining) was detected in MCs across the tissue structures of uterus, myometrium, basal and functional endometrium, and during both the proliferative and secretory phases of the menstrual cycle. (Proliferative n=5, Secretory n=5).

The uterus is an oestrogen target organ and detailed immunohistochemical studies conducted on menstrual cycle staged sections of endometrial tissue by ourselves ( Bombail *et al*., 2008; Critchley *et al*., 2001) and others ( Mylonas *et al*., 2004; Snijders *et al*., 1992) have documented cell and phase-dependent expression of both isoforms of the oestrogen receptor (ERα, ERβ). In the current study, in line with expectation, we identified ERα positive stromal and epithelial cells in the endometrium and stromal fibroblasts in the myometrium ( Supplementary Figure 4); however, although tryptase-positive cells were readily detected in the basal endometrium and myometrium, none of these had detectable ERα protein in their nuclei ( Supplementary Figure 4). In contrast, immunopositive staining for ERβ protein was present in multiple cell types, including stromal fibroblasts and endometrial epithelial cells, as well as tryptase-positive (green cytoplasm) MCs in both the functional and basal regions of the endometrium and throughout the myometrium of the uterus ( Figure 5, arrows). The results obtained with antibody directed against the progesterone receptor mirrored those of ERα, with no evidence of PR-positive MCs ( Supplementary Figure 5).

### Uterine mast cells are immunopositive for the glucocorticoid receptor

**Figure 6.**
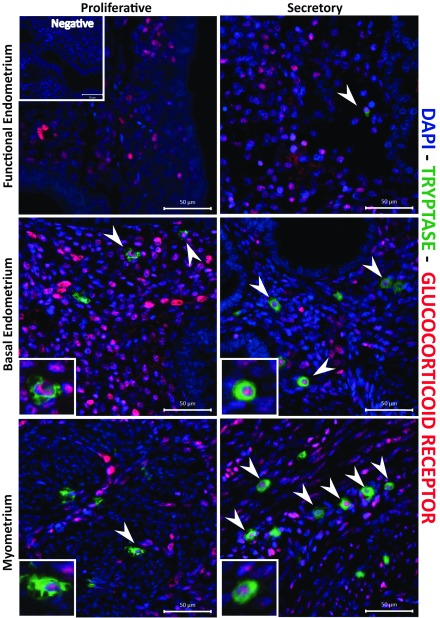
Uterine mast cells in the endometrium and myometrium are immunopositive for glucocorticoid receptor. Mast cell nuclear glucocorticoid receptors (GR; red staining) was detected during the proliferative and secretory phase, throughout the myometrium, functional and basal endometrial and layers. The images are representative of results in proliferative (n=5) and secretory (n=5) phase samples.

We have previously identified GR in multiple cells within the endometrium, including endothelial cells and immune cells ( Rae *et al*., 2009; Thiruchelvam *et al*., 2016), complemented by evidence that enzymes capable of the biosynthesis of cortisol, the natural ligand for GR, are present in the tissue ( Thiruchelvam *et al*., 2016). In the present study, immunostaining for GR showed it was expressed within the stromal fibroblasts and other cells (putative immune cells), as well as being present in the nucleus of tryptase-positive cells in both endometrium and myometrium (arrowheads, Figure 6).

**Figure 7.**
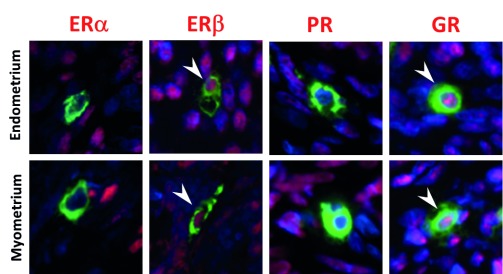
Summary immunoexpression of steroid receptors in human uterine mast cells. Uterine mast cells are immunopositive for ERβ and GR, and immunonegative for ERα and PR. (Red staining: ERα, ERβ, PR and GR; green staining: tryptase). Arrowheads point to nuclei that have immunopositive (red) staining for ERβ and GR.

In summary, we detected co-immunoexpression of both the beta isoform of ER and GR in the nuclei of uterine MCs (tryptase positive staining in their cytoplasm), but no evidence of immunoexpression of ERα or PR. A photomontage of representative sections stained for each of the receptors is provided in Figure 7.

TPSAB1 and CMA1 CT values for qRT-PCR Click here for additional data file. Copyright: © 2017 De Leo B et al. 2017 Data associated with the article are available under the terms of the Creative Commons Zero "No rights reserved" data waiver (CC0 1.0 Public domain dedication).

## Discussion

This study has shed new light both on the phenotype of endometrial and myometrial MCs, as well as revealing the potential that they might respond *in situ* to both oestrogens and glucocorticoids. MCs are known to arise from progenitors in the bone marrow, but adapt to their mature phenotype depending upon the tissue microenvironment in which they mature. To date, uterine MCs have received little attention compared to other immune cell populations, such as uNK cells and macrophages ( Gibson *et al*., 2015; Henderson *et al*., 2003; Thiruchelvam *et al*., 2013). For example, detailed analysis of immune cell populations in endometrium show cyclical variations in their numbers of immune cells, with a notable rise in uNK cells during the secretory phase, a rise in numbers of neutrophils at the start of menstrual tissue breakdown and the largest numbers of macrophages detected during the menstrual phase (reviewed in Maybin & Critchley, 2015).

Analysis of mRNAs encoding proteases expressed by MCs has not previously been reported in endometrial tissue homogenates. We found that the concentrations of tryptase and chymase mRNAs in our samples did not vary significantly between different phases of the menstrual cycle. These results would be consistent with previous reports that MC numbers vary little throughout the menstrual cycle ( Salamonsen *et al*., 2002). We speculate that these results may reflect the long life span of tissue resident MCs, with some studies reporting that MCs have a lifespan of weeks to months ( Kiernan, 1979; Padawer, 1974). As tryptase and chymase mRNAs are constitutively expressed by MCs ( Pejler *et al*., 2007), it is unsurprising they may remain unchanged if cell numbers are fairly constant.

Traditionally, human MCs are classified according to different phenotypes depending on their expression of tryptase and/or chymase in cellular granules ( Irani *et al*., 1986). In line with expectations based on MC phenotype in multiple tissues, we readily detected both tryptase positive and chymase positive cells within both the endometrium and myometrium. Using immunofluorescence, we were able to co-stain for tryptase and chymase in the same cells revealing populations of cells that were of MC _T_ and MC _TC_ phenotypes. Weidner & Austin (1993) were the first to report the existence of a chymase positive/tryptase negative (MC _C_) population of MCs in skin, lung and bowel. MC _C_ have been detected by immunofluorescence in the airway and gastrointestinal tract, reported as being 12% of the MC population in human bronchi and 16.8% bowel submucosa. The current study provides the first evidence for the presence of a chymase positive subpopulation of MCs that did not contain tryptase. This complements and extends a previous study that stained parallel tissue sections with antibodies directed against tryptase and chymase ( Jeziorska *et al.*, 1995), identifying both MC _T_ and MC _TC_, and increases both our understanding of the location and phenotypic heterogeneity of MCs in the uterus.

Results in this study showed the phenotype of the uterine MCs varied between the different tissue layers of the uterus. Confirming previous studies, MC _TC_ were predominantly resident in the basal endometrium and in the myometrium and MC _T_ were found in functional endometrium. The rare MC _C_ type was detected in the basal endometrium and myometrium and completely absent in the functional layer. These findings reinforce the principle that tissue specific phenotypes of MCs can exist within different regions within the same organ. It is already well known that the functional and basal compartments of the endometrium and myometrium vary with regard to cellular composition and cytokine/chemokine concentrations. Interestingly the region of the tissue where MCs appeared most abundant were close to the endometrial-myometrial junction. Previous studies have demonstrated that a large number of CD34 ^+^ MC progenitor cells reside in this area of the tissue and that their numbers are independent of phase of the menstrual cycle ( Cho *et al*., 2004; Mai *et al*., 2008). Finding MCs in close proximity to smooth muscle fibres would be consistent with this cell type being a key source of stem cell factor, and a vital mediator for MC maturation and survival ( Zhang *et al*., 1996).

The activation state of uterine MCs was also explored during the present study. Previously, endometrial MCs were reported to degranulate during the secretory phase, at a time when the tissue is in an oedematous state. This observation was based on detection of extracellular tryptase during oedema and weak intracellular tryptase staining detectable during the proliferative phase ( Jeziorska *et al*., 1995). In this study, endometrial activation and the degranulation of MCs was documented during the early and mid-secretory stages of normal uterine tissue with detection of tryptase and chymase in the extracellular matrix. A ‘recovery’ state, characterised by weak immunostaining, was observed in tissue collected from patients during the proliferative ( Supplementary Figure 1), late secretory ( Supplementary Figure 3) and menstrual ( Figure 4) stages. Within the myometrial compartment, MCs appeared to be in a ‘resting’ state during the proliferative phase ( Supplementary Figure 1). Interestingly, in the myometrium, MCs appeared to be ‘activated’ with detection of immunopositive staining for chymase diffuse and spread beyond the margins of the individual cells during both mid secretory and menstrual phases ( Figure 3 and Figure 4). We speculate that this might suggest a potential role for MC derived proteases in regulation of arteriole sprouting at early/mid secretory phases. A previous study also suggested the release of granules may play a role in smooth muscle contraction during menses ( Sivridis *et al*., 2001) and our findings would be consistent with this suggestion.

Although, other authors have previously demonstrated a direct effect of female sex hormones on MC behaviour, activation and migration, in those studies they cited activation of MCs via ERα and PR ( Jensen *et al*., 2010; Zaitsu *et al*., 2007). The study by Engemise *et al.* (2011) examined the number of mast cells, identified by toluidine blue, in the endometrium of 28 women with endometriosis before and after insertion of a Mirena coil secreting the progestogen levonorgestrel. They reported a reduction in mast cell number after 6 months but found no evidence of staining for PR or ERα. In the current study, based on detailed immunohistochemical analysis, using previously validated antibodies directed against oestrogen receptor subtypes ( Critchley *et al*., 2002; Henderson *et al*., 2003), we found novel evidence for immunoexpression of ERβ, but no evidence of expression of ERα. These results mirror the oestrogen receptor phenotype of both uNK ( Gibson *et al.*, 2015; Henderson *et al.*, 2003)) and macrophages ( Thiruchelvam *et al*., 2013). This observation is also supported by the activation of uterine MCs during the secretory phase at a time in the menstrual cycle when intracrine biosynthesis of oestradiol has been shown to activate ERβ positive uNKs ( Gibson *et al*., 2015).

In other studies, we have shown that in endometrial tissue expression of ERβ often parallels that of GR with co-expression in both uNK ( Henderson *et al*., 2003) and endothelial cells ( Critchley *et al*., 2002). In the current study, nuclear GR was detected in MCs in the functional, basal endometrium and myometrium. In the same samples, GR protein was also detected in endometrial stroma and smooth muscle fibres during the proliferative phase, but its expression was reduced during the progesterone-dominant secretory phase, a result which was in agreement with previous reports ( Bamberger *et al*., 2001; Henderson *et al*., 2003). Several studies have reported that glucocorticoids may have an indirect anti-inflammatory impact on MCs, with the postulated mechanism being a reduction of stem cell factor production by fibroblasts ( Da Silva *et al*., 2002). Alternatively, they may also have direct impacts by reducing IgE binding to the FcεRI receptors, thereby down regulating the expression of these receptors on the cell membrane of the MCs and inhibiting MC degranulation *in vitro* ( Finotto *et al*., 1997; Yamaguchi *et al*., 2001; Zhou *et al*., 2008). Prior to the current study, the only report of expression of GR in human MCs was from Oppong *et al*. (2014). In their study, they localized GR to the plasma membrane in the RBL-2H3 MC line. The current study is the first to demonstrate that uterine MCs are immunopositive for nuclear GR. A glucocorticoid rich environment would favour activation of GR, with shuttling of ligand activated receptor from the cytoplasm towards the nucleus ( Phuc Le *et al*., 2005). This observation would be consistent with expression 11-β hydroxysteroid dehydrogenase enzymes within the uterus, resulting in a cortisol rich microenvironment ( Gibson *et al*., 2013; McDonald *et al.*, 2006).

In summary, our study confirms that MCs are members of the leukocyte population of the human uterus, and that they are most abundant in the myometrial and basal endometrial compartments. Whilst uterine MCs predominantly belong to the classic MC subtypes: tryptase positive/chymase negative (MC _T_) and tryptase/chymase positive (MC _TC_), a rare third subtype (MC _C_) was also identified in the uterus for the first time. We demonstrated that endometrial and myometrial MCs are immunopositive for both ERβ and GR, demonstrating that, like other immune cells present in the endometrium (uNK, macrophages), they may be a target for the *direct* actions of oestrogens and glucocorticoids, which are both synthesised within the endometrial tissue microenvironment. This study provides a framework for furture studies on the role of MCs in endometrial and myometrial disorders, including conditions associated with increased pain, such as endometriosis.

## Data availability

The data referenced by this article are under copyright with the following copyright statement: Copyright: © 2017 De Leo B et al.

Data associated with the article are available under the terms of the Creative Commons Zero "No rights reserved" data waiver (CC0 1.0 Public domain dedication). 

Dataset 1: TPSAB1 and CMA1 CT values for qRT-PCR. doi, 10.5256/f1000research.11432.d160468 ( De Leo *et al.*, 2017).

## Ethical approval

Lothian Research Ethics Committee (LREC) approval was granted and written patient consent was obtained prior to tissue collection by dedicated research nurses (approval numbers, 10/S1402/59 and 16/ES/0007).

## Supplementary material

**Supplementary Table 1. Details of patients, cycle stage, diagnosis and use of individual samples in different experimental protocols.**

.

**Supplementary Table 2. Details of antibodies used for immunofluorescence.**

.

**Supplementary Figure 1. Immunolocalisation of tryptase and chymase in proliferative phase endometrium.** Single fluorescence channels show the different mast cell subtypes in a uterine “full thickness” section during proliferative phase of the menstrual cycle. The myometrial compartment shows three different mast cell subtypes: tryptase single positive, chymase single positive and tryptase and chymase double positive. Basal endometrial mast cells are tryptase single positive and double positive, instead functional endometrium MCs are fewer in number and show a tryptase only phenotype. The MC activation profile during proliferative phase looks quiescent; proteases are retained in the cytoplasm. (n=4) (White triangles: MCTC cells; white Vs: MCT cells; white arrows: MCC cells).

.

**Supplementary Figure 2. Mast cell subtypes and activation state during the early secretory phase.** Single fluorescence channels show the different mast cell subtypes in a uterine full thickness section during the early secretory phase. Myometrial compartment shows three different mast cell subtypes: tryptase single positive, chymase single positive and tryptase and chymase double positive. Basal endometrial mast cells are tryptase single positive and double positive, instead functional endometrium MCs are fewer in number and show a chymase negative phenotype. MCs during the early secretory phase appeared to be activated, releasing both proteases from the cytoplasm. (n=4) (White triangles: MCTC cells; white Vs: MCT cells; white arrows: MCC cells).

.

**Supplementary Figure 3. Mast cell subtypes and activation state during the late secretory phase.** During the late secretory phase, MCs are identified as tryptase single positive and weakly double positive (MCTC). In both the endometrial layers MCs showed a strong tryptase only phenotype. MCs during the late secretory phase appeared to be activated only in the myometrial compartment, releasing tryptase and retaining chymase in the cytoplasm. (n=3) (White triangles: MCTC cells; white Vs: MCT cells).

.

**Supplementary Figure 4. Immunoexpression of ERalpha (ERα) was detected in multiple cell types within the endometrium and myometrium but not in the nuclei of tryptase-positive mast cells (MCs).** Double immunofluorescence showed no ERα immunoexpression (red staining) in the nuclei of uterine MCs (green staining). MCs were noted to be immunonegative in all uterine layers and across the phases of the menstrual cycle. Myometrial, stromal andepithelial cells showed expression for ERα, as expected (Proliferative n=5, Secretory n=5).

.

**Supplementary Figure 5. Mast cells and progesterone receptor (PR) immunoexpression in the human uterus.** Mast cells were demonstrated to be immunonegative for PR expression (red staining), across “full thickness” uterine sections and during both the proliferative and secretory phase. (Proliferative n=5, Secretory n=5).
