# Supplementary material for: Immunoprofiling of human uterine mast cells identifies three phenotypes and expression of ERβ and glucocorticoid receptor
Source: F1000Res. 2017 Jun 22;6:667. Originally published 2017 May 12. [Version 2] doi: 10.12688/f1000research.11432.2 (PMC5461902; doi:10.12688/f1000research.11432.2)
Supplement: Supplementary file 2 [file f1000research-6-12942-s0001.tgz › cfb2b140-9d07-46fa-b8ad-f84cd3f33fc1.docx]

Supplementary Table 1: Details of patients, cycle stage, diagnosis and use of individual samples in different experimental protocols.

Tissues were collected under ethical approval number 10/S1402/59 and 16/ES/0007 from Lothian Research Ethics Committee. n.a.: not applicable, TAH: total abdominal hysterectomy, STAH: subtotal abdominal hysterectomy, VagHyst: vaginal hysterectomy, PFR: pelvic floor repair, LAVH: laparoscopically assisted vaginal hysterectomy, LNG-IUS: levonorgestrel-releasing intrauterine system, LapSterRev: laparoscopic sterilization reversal, IF: double immunofluorescence.

| Unique No | Serum E_2_ (pg/ml) | Serum P_4_ (ng/ml) | H&E Stage | Age | Average Menstrual cycle length | Surgical procedure | Sample Application |
| --- | --- | --- | --- | --- | --- | --- | --- |
| 5433 | 103.57 | 2.47 | Proliferative | 39 | 29 | TAH | IF |
| 7815 | 3.3 | <1 | Proliferative | 42 | n.a. | Endometrial sampling | RNA |
| 5323 | 107 | 0.94 | Proliferative | 45 | 30 | TAH | IF |
| 5491 | 185 | 9.42 | Proliferative | 40 | 22 | TAH | RNA |
| 7680 | 215 | <3 | Proliferative | 36 | 28 | TAH | IF |
| 7151 | 245 | 2.7 | Proliferative | 36 | n.a. | Insert LNG-IUS | RNA |
| 7541 | 295 | 4.02 | Proliferative | 33 | 31.5 | LapSter | RNA |
| 5342 | 318 | 2.6 | Proliferative | 45 | 21 | VagHyst | IHC/IF |
| 5144 | 357 | 1.8 | Proliferative | 41 | 30 | LapSterRev | RNA |
| 5021 | 428 | 6.4 | Proliferative | 42 | 30 | LapSter | RNA |
| 7728 | 444 | <3 | Proliferative | 42 | 30 | Hysteroscopy | RNA |
| 5612 | 472 | 2.33 | Proliferative | 37 | 28 | LapSter | RNA |
| 5312 | 565 | 5.49 | Proliferative | 42 | 21 | TAH | IF |
| 5007 | 570 | 3.7 | Proliferative | 44 | 27 | TAH | IF |
| 7854 | 571 | 10.2 | Proliferative | 47 | 21 | TAH | IF |
| 7903 | 628 | 3.2 | Proliferative | 40 | 23.5 | TAH | IF |
| 7283 | 679.57 | 4.46 | Proliferative | 40 | 28 | LapSter | RNA |
| 7115 | 921 | 7.89 | Proliferative | 44 | 28 | TAH | IF |
| 5201 | 943 | 3.24 | Proliferative | 39 | 26.5 | LapSter | RNA |
| 5616 | 1178 | 2.2 | Proliferative | 44 | 26.5 | LapSter | RNA |
| 5074 | 988 | 34.1 | Early Secretory | 35 | 28 | LapSter | RNA |
| 5158 | 604 | 42.5 | Early Secretory | 39 | 26 | LapSter | RNA |
| 5037 | 592 | 46.1 | Early Secretory | 32 | 30.5 | LapSter | RNA |
| 5067 | 514 | 22.8 | Early Secretory | 44 | 28 | Polypectomy | RNA |
| 5014 | 407 | 44.4 | Early Secretory | 36 | 28 | LapSter | RNA |
| 7210 | 289.28 | 89.93 | Early Secretory | 33 | 28 | VagHyst &PFR | IF |
| 7882 | 338 | 28.5 | Early Secretory | 43 | 28 | VagHyst &PFR | IF |
| 5300 | 398 | 63 | Early Secretory | 35 | 30 | Labial Cyst | RNA |
| 7248 | 549.91 | 88 | Early to Mid Secretory | 32 | 28 | TAH | IF |
| 5211 | 502 | 43.2 | Early to Mid Secretory | 43 | 24.5 | STAH | IF |
| 3155 | 351 | 23.1 | Early to Mid Secretory | 32 | 28 | TAH | IF |
| 6412 | 460 | 32.5 | Mid Secretory | 39 | 30 | Ovarian Cystectomy | RNA |
| 5624 | 457 | 25.83 | Mid Secretory | 30 | 28 | Polypectomy | RNA |
| 7384 | 331 | 83.5 | Mid Secretory | 42 | 24.5 | TAH | IF |
| 5690 | 323 | 38.6 | Mid Secretory | 39 | 27.5 | TAH | RNA |
| 7730 | 312 | 43.2 | Mid Secretory | 39 | 29 | Polypectomy | RNA |
| 7941 | 271 | 33.4 | Mid Secretory | 46 | 28 | TAH | RNA |
| 6359 | 203.3 | 16.63 | Mid Secretory | 42 | 28 | LapSter | RNA |
| 5607 | 38.2 | 7.09 | Mid to Late Secretory | 38 | 25 | TAH | IF |
| 5743 | 132 | 6.4 | Late Secretory | 47 | 28 | TAHBSO | IF |
| 5135 | 602 | 14.7 | Late Secretory | 44 | 28 | TAHBSO | IF |
| 7817 | 8.4 | 2.3 | Menstrual | 42 | 29.5 | Endometrial sampling | RNA |
| 5316 | 371 | 7.54 | Menstrual | 40 | 28 | LAVH | RNA |
| 7229 | 175.87 | 2.55 | Menstrual | 40 | 28 | TAH | IF |
| 7909 | 242 | <3 | Menstrual | 35 | 24.5 | STAH | IF |
| 5609 | 196 | 8.21 | Blood Clot Only | 38 | 30 | Ovarian Cystectomy | RNA |
