# Supplementary material for: Immunoprofiling of human uterine mast cells identifies three phenotypes and expression of ERβ and glucocorticoid receptor
Source: F1000Res. 2017 Jun 22;6:667. Originally published 2017 May 12. [Version 2] doi: 10.12688/f1000research.11432.2 (PMC5461902; doi:10.12688/f1000research.11432.2)
Supplement: Supplementary file 3 [file f1000research-6-12942-s0002.tgz › 573aa927-63f3-4c29-98be-da329fdab93e.docx]

**Supplementary Table 2. Details of antibodies used for immunofluorescence.**

| Primary Antibody | **Species raised** | **Source** | **Clone** | **Concentrations** | **Dilution** | **Secondary Antibody** |
| --- | --- | --- | --- | --- | --- | --- |
| Tryptase | Rabbit | Abcam (ab134932) | EPR8476 | 3.428 mg/ml | 1:300 | 1:200 - Goat anti-rabbit peroxidase (ab7171) |
| Chymase | Mouse | AbSerotec (MCA1930T) | CC1 | 1.0mg/ml | 1:5000 | 1:200 - Goat anti-mouse peroxidase (ab6823) |
| Oestrogen Receptor α (ERα) | Mouse | Vector (VP-614) | 6F11 | 7.5mg/ml | 1:200 | 1:200 - Goat anti-mouse peroxidase (ab6823) |
| Oestrogen Receptor β (ERβ) | Mouse | AbSerotec (MCA1974GA) | PPG5/10 | 1.0mg/ml | 1:200 | 1:200 - Goat anti-mouse peroxidase (ab6823) |
| Progesterone Receptor (PR) | Mouse | Novacastra (NCL-PGR-312) | 16 | 1.0-8.0mg/ml | 1:3000 | 1:200 - Goat anti-mouse peroxidase (ab6823) |
| Glucorticoid Receptor (GR) | Mouse | Novacastra (NCL-GCR) | 4H2 | 25μg/ml. | 1:100 | 1:200 - Goat anti-mouse peroxidase (ab6823) |
