# Supplementary figures and images for: Immunoprofiling of human uterine mast cells identifies three phenotypes and expression of ERβ and glucocorticoid receptor
Source: F1000Res. 2017 Jun 22;6:667. Originally published 2017 May 12. [Version 2] doi: 10.12688/f1000research.11432.2 (PMC5461902; doi:10.12688/f1000research.11432.2)

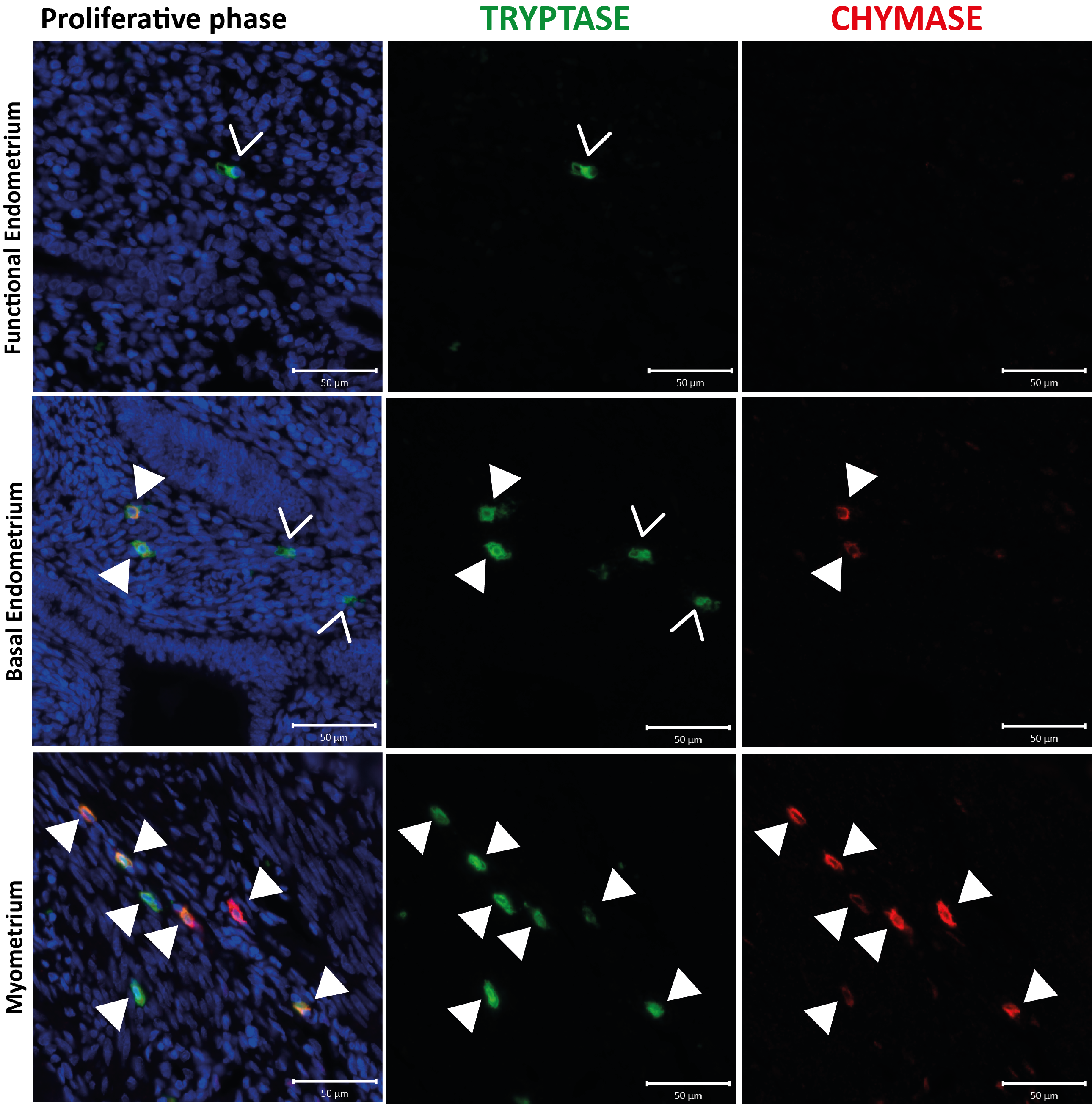

Supplement: Supplementary file 4 [file f1000research-6-12942-s0003.tgz › e92f3278-b279-4abf-a379-ab5148203260.png]

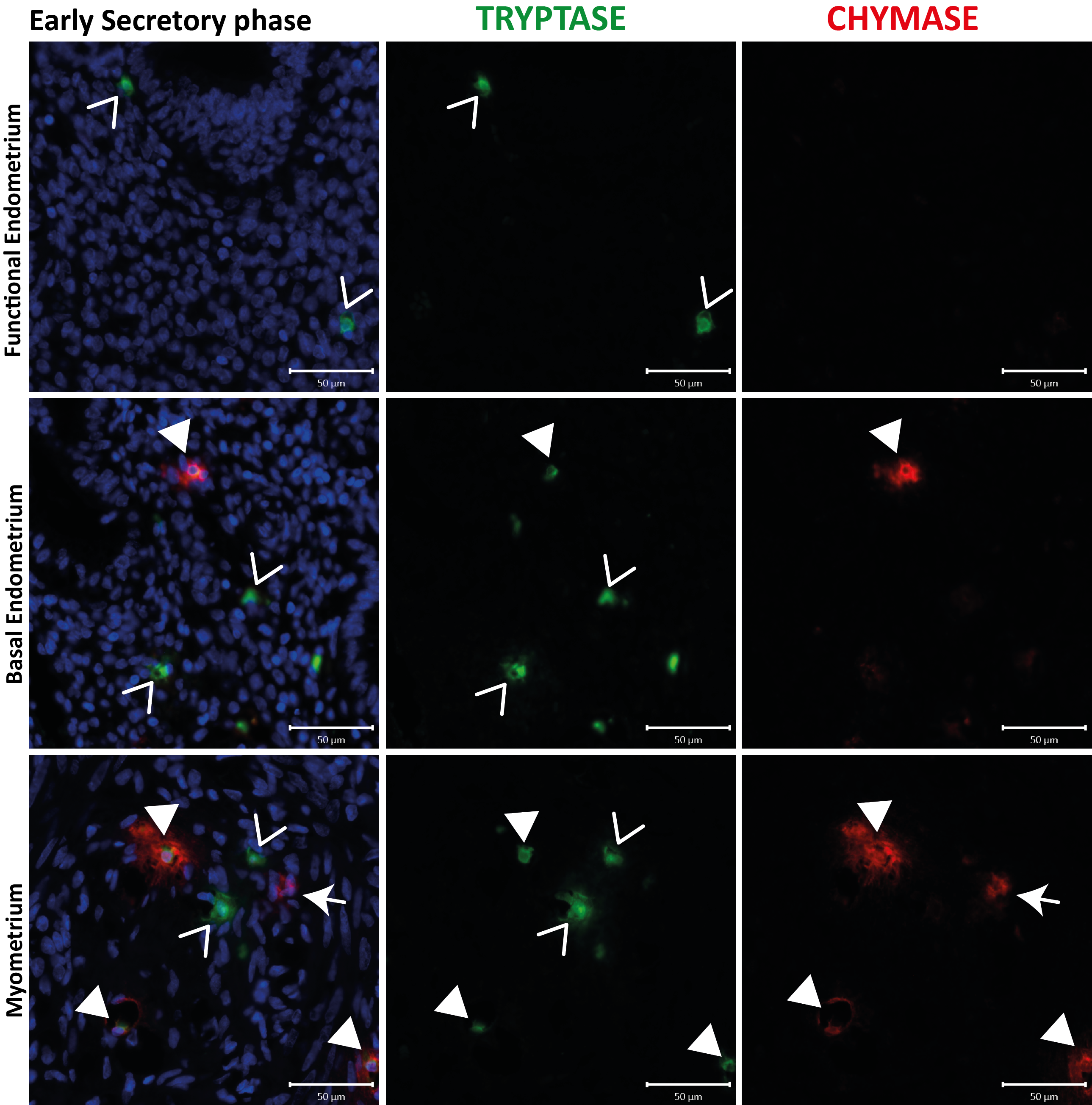

Supplement: Supplementary file 5 [file f1000research-6-12942-s0004.tgz › 9eadcbf3-1179-48d4-bd5f-f5b7eac49c32.png]

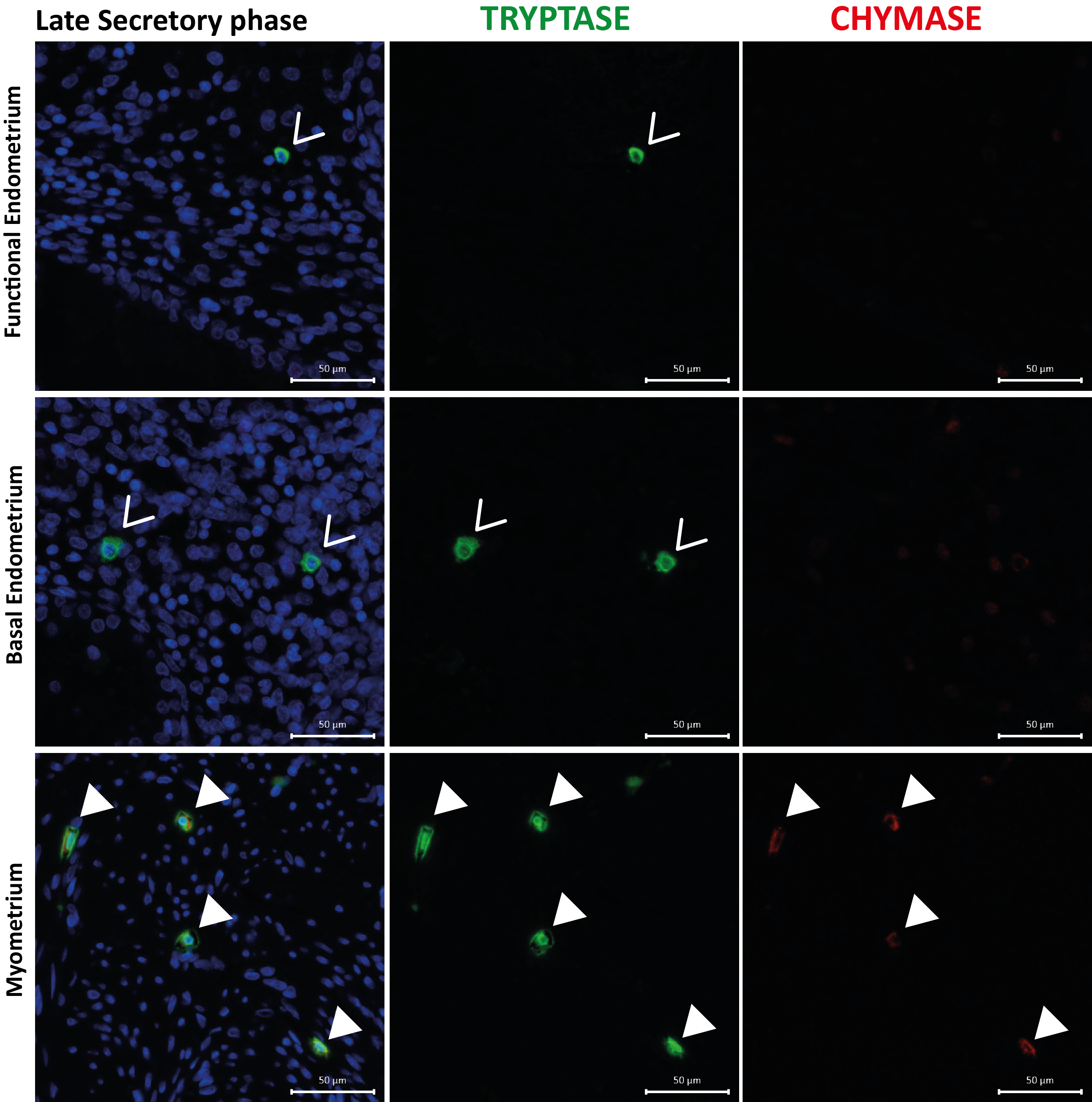

Supplement: Supplementary file 6 [file f1000research-6-12942-s0005.tgz › b095b730-90ea-4fcf-b0e4-b453c76e8f4e.png]

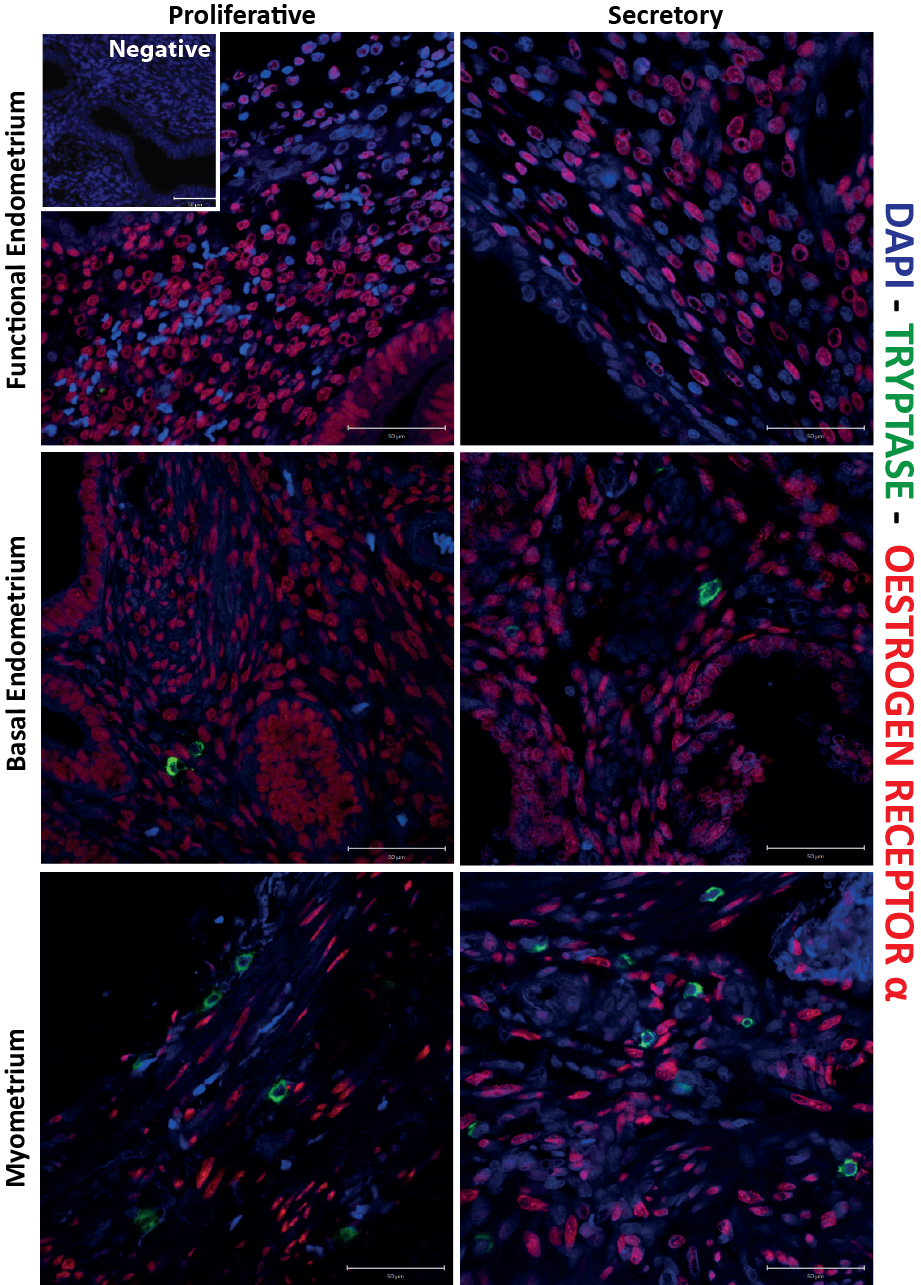

Supplement: Supplementary file 7 [file f1000research-6-12942-s0006.tgz › 54aab5a7-b173-422c-9ea4-87c331033dad.png]

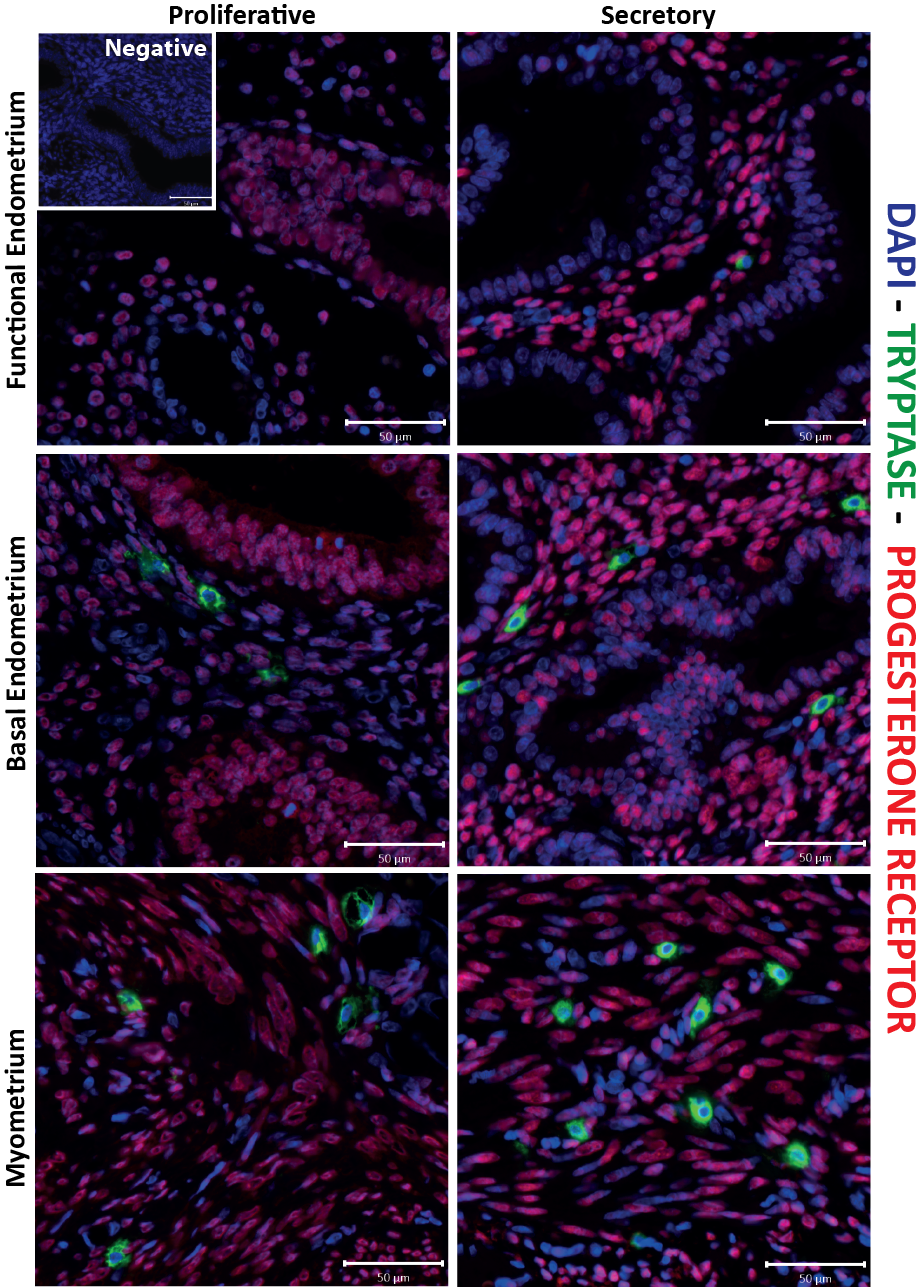

Supplement: Supplementary file 8 [file f1000research-6-12942-s0007.tgz › 785a807e-3494-46b6-9c8b-86b3e3bf9fde.png]
