# Supplementary material for: Immunoprofiling of human uterine mast cells identifies three phenotypes and expression of ERβ and glucocorticoid receptor
Source: F1000Res. 2017 Jun 22;6:667. Originally published 2017 May 12. [Version 2] doi: 10.12688/f1000research.11432.2 (PMC5461902; doi:10.12688/f1000research.11432.2)
Supplement: Peer review history

# Peer review: Referee response for version 1

**DOI:** 10.5256/f1000research.12343.r23114

**Published:** 2017-05-31

**Authors:** Areege M. Kamal

**Affiliations:** 1 The National Centre for Early Detection of Cancer, Oncology Teaching Hospital, Baghdad Medical City, Baghdad, Iraq; 2 University of Liverpool, Liverpool, UK

This is an observational, descriptive study describing the enzymatic and hormonal phenotype of the mast cells in full thickness normal human endometrium and the adjacent myometrial layers. The data from the modest sample size presented in the manuscript confirms previous reports of spatio-temporal distribution of MC and their phenotype throughout the menstrual cycle; highlighting the novel finding, the glucocorticoid receptor expression in these cells. The authors have also examined the GR expression in the context of the expression of a selected panel of other steroid receptors. The manuscript is well written and presented; however, there are some points detailed below which warrants further consideration/ clarification.

**General comments:**

 The conventional histopathological description of the endometrial layers as endometrial basalis layer and functionalis layer, or stratum basalis or stratum functionalis throughout the manuscript may provide more clarity than the inconsistent use of less conventional descriptive terms of basal compartment etc. in the present MS.

 Referral to some pivotal previous references could make the manuscript more comprehensive, such as Engemise SL *et al,* Eur J Obstet Gynecol Reprod Biol., 2011; Mori A *et al.*, Hum Reprod., 1997; Drudy L et al.,  Eur J Obstet Gynecol Reprod Biol., 1991

 The description of the genes *TPSAB1* and *CMA1 *first appear in the results section but should be stated at the initial mention for clarity.  

**Methods:**

1. Authors have analysed the *TPSAB1* and *CMA1 *mRNA expression by relative standard curve using a tonsil mRNA as a reference. What is the reason for this and why was this method utilised in place of the usual comparative Ct (delta delta Ct) method? The reasons need to be explained with reference to the more widely known method.
2. In dual IHC, was the 2RT antibody incubation for 16h? This appears to be relatively longer than the usual protocols. Did the authors use a quantification method to assess the immunolocalisation of the proteins of interest?

 

**Results:**

1. The total number of samples mentioned in the Methods is 46, yet there is inconsistent numbers used at different aspects of the analysis. Particularly in the paragraph describing steroid receptors co-stained with tryptase, the number of samples stated in the legends of referenced figures does not add up to 20 as mentioned in the text.
2. The authors present data on the steroid receptor expression in tryptase-positive uterine mast cells why did they not present the data for the same in the chymase-positive cells? It is noticeable that not all the MC were ERβ+, this might be associated with chymase expression?
3. Data in Table 1 could be more informative, with quantification of the expression and statement if this refers to the exact endometrial layer; basalis or functionalis.
4. In Figure 6, GR+ MCs seems to be activated. Is this pertinent to all the samples? Or is it phase specific phenomenon?

 

**Discussion:**

1. Page 10, “We found that the **concentrations** of tryptase and chymase mRNA  ”, should be replaced with “levels”
2. The statement “As tryptase and chymase are constitutively expressed by MCs (Pejler *et al*., 2007), it is unsurprising they may remain unchanged if cell numbers are fairly constant.” Need to be updated with reference to activated MC releasing the tryptases to the extracellular compartment, but the extracellular compartment is part of the whole endometrial lysate that has been examined so overall levels are not expected to change anyway.
3. The reference to some other papers that agree and disagree with their work (mentioned above), authors may also acknowledge the small sample size as a limitation of their study.

I have read this submission. I believe that I have an appropriate level of expertise to confirm that it is of an acceptable scientific standard.

# Response

**Published:** 2017-06-17

**Authors:** Philippa Saunders

**Affiliations:** MRC Centre for Inflammation Research, University of Edinburgh, UK

We thank Dr Kamal for her comments on our paper which we have edited to address some of her concerns.

Specific responses:

We have now referenced the small study by Mori *et al* (1997) in the Introduction and the study by Engemise *et al* in the Discussion.

We have changed the text of the Introduction so that the abbreviations for TPSAB1 and CMA1 are introduced during the first mention of their importance in defining the mast cell phenotype.

Methods.

The text describing the samples has been changed to highlight the patient information provided in Suppl Table 1 and clarify that although 46 patients were recruited during this study their samples were split between RNA (n=29) and fixation for immunostaining (n=26).

Analysis methods for RTPCR data: Although the delta delta CT method is one of the most popular it is based on comparable amplification efficiencies of the endogenous control and gene of interest. As determined by the standard curves the efficiencies of the TPSAB1 and CMA1 were not suitable for analysis by this method and therefore a more appropriate method was the relative standard curve method. The text in M&M has been revised to make this clearer.

See: Bustin SA, Benes V, Garson JA etal, the MIQE guidelines: Minimum Information for Publication of Quantitative Real-Time PCR experiments, Clinical Chemistry 55:4 611-622 (2009)

These methods are also described in detail in Applied Biosystems manual online

http://www6.appliedbiosystems.com/support/tutorials/pdf/performing_rq_gene_exp_rtpcr.pdf

The tonsil sample was chosen as a positive control as this tissue has mast cells which contain tryptase and chymase, the RNA sample was purchased from a commercial supplier and we chose this tissue because we were able to access fixed material from our hospital to use for control experiments to validate the antibodies.

For dual immunostaining primary antibodies were applied to sections overnight (16h) and incubated in a fridge - secondary antibodies were applied for 1h at room temperature. This is a standard method used in our laboratory and in our hands it leads to lower rates of non-specific background staining than application of primary antibody at room temperature. We did not quantify the fluorescent staining.

Results.

The limitations on the amount of individual fixed samples meant that not all of the fixed samples (n=20) were stained with all antibodies and this is reflected in the numbers stated in the figure legends.

MCs were considered as ERbeta positive only if the entire nucleus was identified in the section (as highlighted with white arrowheads). In this study the low number of chymase only MCs meant we were not able to conduct a comprehensive staining for ERbeta.

In the summary Table 1 we decided to combine the results from the functional and basal layers because the mast cells were very rare in the former and we wanted to provide an overview to complement the more comprehensive data shown in the individual photographs.

Our interpretation of the data related to GR positive cells (as illustrated in Figure 3, 6 panels) is that the tryptase positive mast cells that were GR+ appeared to be in resting state i.e. the tryptase appeared to be confined to the cytoplasmic area around the nucleus.

Discussion.

We used a standard curve method so 'concentration' is more appropriate than 'levels' which is a common term when the delta CT method is used.

We have changed the text to make it clearer that this paragraph is discussing mRNAs and hence protein release is not relevant to the argument about constitutive expression.

# Peer review: Referee response for version 1

**DOI:** 10.5256/f1000research.12343.r22759

**Published:** 2017-05-16

**Authors:** Lois Salamonsen

**Affiliations:** 1 Hudson Institute of Medical Research, Clayton, VIC, Australia

This article examines in detail the phenotypes of mast cells in the uterus – depending on their tryptase/chymase content and their steroid hormone receptor status. The work is solid, and well presented. The methods are well described so that others can validate the data. While tryptase/chymase phenotypes have previously been described across the menstrual cycle (referenced), the information regarding a tryptase-/chymase+ phenotype is new, as is the steroid receptor phenotypeof ERβ+/GR+/ERα-/PR-  in tryptase positive cells. Where the data particularly varies from that previously published is in the activation status of the mast cells. This variation could be explained by different fixation protocols or different antibodies. Activation is very important as mast cells are only functionally relevant following this release of the potent molecular contents of their granules.

I have read this submission. I believe that I have an appropriate level of expertise to confirm that it is of an acceptable scientific standard.

# Response

**Published:** 2017-06-17

**Authors:** Philippa Saunders

**Affiliations:** MRC Centre for Inflammation Research, University of Edinburgh, UK

We thank Professor Salamonsen for her positive comments: we agree different methods of fixation can affect results in different studies. All the samples in this study were processed according to standard methods approved by our pathologist.
